# Capacity-Speed Relationships in Prefrontal Cortex

**DOI:** 10.1371/journal.pone.0027504

**Published:** 2011-11-23

**Authors:** Vivek Prabhakaran, Bart Rypma, Nandakumar S. Narayanan, Timothy B. Meier, Benjamin P. Austin, Veena A. Nair, Lin Naing, Lisa E. Thomas, John D. E. Gabrieli

**Affiliations:** 1 Radiology, University of Wisconsin-Madison, Madison, Wisconsin, United States of America; 2 Neuroscience Training Program, University of Wisconsin-Madison, Madison, Wisconsin, United States of America; 3 School of Medicine and Public Health, University of Wisconsin-Madison, Madison, Wisconsin, United States of America; 4 Center for Brain Health, University of Texas-Dallas, Dallas, Texas, United States of America; 5 Department of Neurology, Yale University, New Haven, Connecticut, United States of America; 6 Cardiovascular Research Center, University of Wisconsin-Madison, Madison, Wisconsin, United States of America; 7 Departments of Emergency Medicine, Brigham & Women's Hospital and Massachusetts General Hospital, Boston, Massachusetts, United States of America; 8 Department of Brain and Cognitive Sciences, Massachusetts Institute of Technology, Cambridge, Massachusetts, United States of America; University of Groningen, Netherlands

## Abstract

Working memory (WM) capacity and WM processing speed are simple cognitive measures that underlie human performance in complex processes such as reasoning and language comprehension. These cognitive measures have shown to be interrelated in behavioral studies, yet the neural mechanism behind this interdependence has not been elucidated. We have carried out two functional MRI studies to separately identify brain regions involved in capacity and speed. Experiment 1, using a block-design WM verbal task, identified increased WM capacity with increased activity in right prefrontal regions, and Experiment 2, using a single-trial WM verbal task, identified increased WM processing speed with increased activity in similar regions. Our results suggest that right prefrontal areas may be a common region interlinking these two cognitive measures. Moreover, an overlap analysis with regions associated with binding or chunking suggest that this strategic memory consolidation process may be the mechanism interlinking WM capacity and WM speed.

## Introduction

Over fifty years ago, George A. Miller was “persecuted” by a magical number which he believed could underlie the basis of human short-term memory capacity. His “Seven, Plus or Minus Two” theory was a scientific turning point in the study on the limits of human memory that has lead to a vast amount of research aimed at demystifying this number. But just as *capacity* gained attention in the field of cognition, so did *processing speed* become a focus of scientific intrigue. In 1966, Saul Sternberg quantified limits on the speed of retrieval of items stored in short-term memory [1]. His posited internal serial-comparison process launched a new era in cognitive research aimed at elucidating the mechanism behind processing speed. It wasn't until 1972, however, that speed and capacity were inter-linked through an elegant meta-analysis [2], a study which catalyzed the investigation of speed and capacity as interdependent concepts. Since then, a third and equally important concept in the field of cognitive science has emerged; “Binding” or “chunking” is a strategic memory consolidation process, and it is this concept that is thought to inter-link speed and capacity. The current functional MRI study aims to provide insights into the brain bases behind these three concepts and furthermore show for the first time brain evidence inter-linking these concepts.

Working Memory (WM), the cognitive system that permits temporary information maintenance and manipulation, underlies many higher cognitive functions including text comprehension, reasoning, and problem solving (e.g., [3]). Not surprisingly, WM impairments are observed in a variety of conditions marked by executive dysfunction including neurodegenerative diseases and psychiatric disorders [4], [5]. Moreover, deficits in WM have been proposed to be the major cause of cognitive dysfunction associated with normal aging [6]. Thus, understanding the neural basis of the capacity-speed relationship in WM has the potential to improve the assessment and treatment of cognitive deficits that affect a variety of populations.

WM is typically described in terms of storage capacity and processing speed. WM capacity, which has been thought of as a measure of the allocated processing resource that is utilized for successful performance of higher cognitive tasks (e.g., [7]), is a limited resource that can be flexibly shifted [8]. Capacity has been measured by span tasks that require either brief maintenance (in the case of simple span tasks) or simultaneous maintenance and manipulation processes (in the case of complex span tasks; e.g., [7], [9], [10]). Results from behavioral studies suggest that while simple and complex span tasks share significant proportions of variance, they index different WM components, with simple span tasks reflecting maintenance and complex span tasks reflecting both maintenance and manipulation processes.

WM speed has been thought of as a measure of the rate of information processing that is utilized for successful performance of higher-order cognitive tasks [11]. Sternberg used an item recognition paradigm to estimate this rate by calculating the increase in retrieval time (RT) associated with increasing numbers of to-be-remembered items [1]. The monotonic function relating RT to numbers of to-be-remembered items has suggested an important role for processing speed in WM performance.

WM capacity and WM speed interrelationships have been observed in numerous behavioral studies. Correlations between WM capacity (as indexed by simple span measures), and processing speed (as indexed by simple processing-speed tasks measuring RT) have been consistently observed [2], [11], [12]. Studies of both adult and child development have consistently shown relationships between measures of processing-speed and WM capacity [11], [13]–[15]. These results suggest that WM capacity and speed may be interdependent determinants of performance. Persistent behavioral findings of capacity-speed relationships and neuroimaging studies displaying speed-activation and capacity-activation relations suggest that these two performance indices may be related by a common neural mechanism [9], [16]–[21]. Understanding the brain basis of capacity-speed relations may provide evidence that would elucidate this common mechanism.

One possibility, suggested by previous behavioral [22] and neuroimaging research, is that some individuals may utilize capacity resources by implementing strategic memory consolidation processes (e.g., binding, or chunking of information stored in WM; [16]–[21]). In one study, for instance, participants were required to maintain up to 8 letters over a 12 second retention interval. Those who performed better (i.e., above the median) in the task showed memory-load related prefrontal cortex (PFC) increases in activation during retention compared to those who did not perform as well [19]. Similarly, maintenance-related PFC activation has been reported to increase during longer retention intervals relative to control conditions matched for difficulty [23]. These results suggest that those participants who organize information over the retention interval may effectively reduce WM demand, thereby increasing WM capacity and WM retrieval speed. The result of this increased processing could be increased WM capacity, leading to faster processing speed. Thus, we predicted that individual participants' neural activity during WM maintenance would be related to their WM capacity, as measured by a simple span task (e.g. Digit Span Forward score), and their WM processing speed, as measured by a simple processing-speed task (e.g. item-recognition rate in Sternberg WM paradigm).

In the present study, we carried out two fMRI experiments to elucidate the brain basis of the relationship between WM capacity and speed. *Experiment 1* was conducted in order to elucidate regions related to WM capacity. *Experiment 2* was conducted in order to elucidate regions related to WM speed. We hypothesized that there would be an overlap in brain regions related to capacity and speed which would elucidate this common mechanism between capacity-speed relationships.

**Figure 1:**
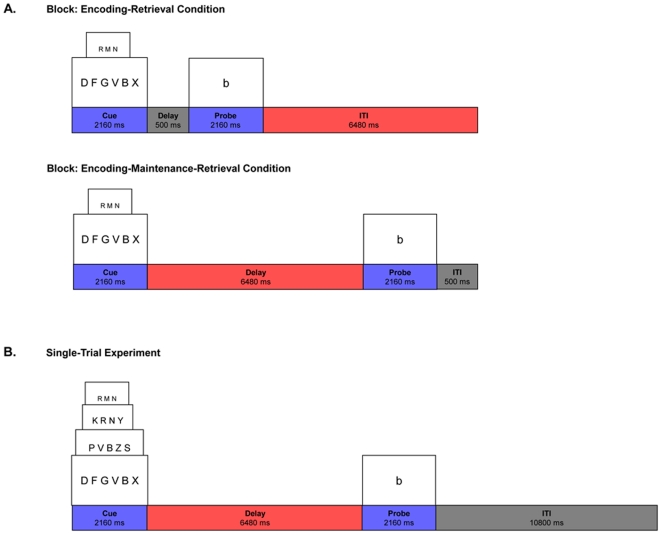
Experimental stimuli. (A) Trial sequence and examples of stimuli in the 3- and 6-letter ER (encoding-retrieval) conditions (upper row) and the 3- and 6-letter EMR (encoding-maintenance-retrieval) conditions (lower row) used in the block design fMRI paradigm (*Experiment 1*). For all trials, participants were asked to remember a target display comprised of 3 or 6 upper-case consonants over a 2160 ms interval. Participants had to maintain letter information for either 500 ms (upper row) or 6480 ms (lower row). On probe trials, participants had 2160 ms to determine whether a single lower-case consonant had been in the target display. A 6480 ms intertrial interval (ITI) was added to the trials with short delays and a 500 ms ITI was added to the trials with long delays in order to equate the time between target displays. (B) Trial sequence and examples of stimuli in the 3, 4, 5, and 6-letter conditions used in the single-trial fMRI paradigm (*Experiment 2*). For all trials, participants were asked to remember a target display comprised of 3, 4, 5, or 6 upper-case consonants over a 2160 ms interval. Participants had to maintain letter information for 6480 ms. On probe trials, participants had 2160 ms to determine whether a single lower-case consonant had been in the target display. A 10800 ms ITI ensued in order to allow for hemodynamic response to return to baseline.

In *Experiment 1*, twelve participants were scanned while performing a Sternberg-type item-recognition task with two memory load conditions (3-letter, 6-letter), with (Encode-Maintain-Retrieve; EMR) and without (Encode-Retrieve; ER) delay in a block design task (Figure 1A). In *Experiment 2*, a different set of twelve participants were scanned using the same EMR task, but with an event-related design so that participants maintained different numbers of letters on different trials (Figure 1B). Prior to scanning, all subjects received a battery of paper and pencil tests including the Digit Span Forward task outside the scanner. The paper and pencil test results indicated relatively equivalent scores for the two groups that participated in *Experiments 1* and *2* (Table S1).

## Methods

All studies were done in full compliance with the guidelines of the Institutional Review Board (Assurance #M1272-02) of Stanford University.

### Experiment 1

#### Participants

Twelve subjects (5 female and 7 male right-handed native-English speakers, mean age = 19.8 yrs) were scanned via functional magnetic resonance imaging (fMRI). All participants were undergraduate students from Stanford University and each provided a written consent which was approved by the Institutional Review Board.

#### Behavioral Task

Subjects performed a Sternberg-type verbal WM task [1] (see Figure 1A). In the encoding (E) phase of each trial, participants encoded three or six uppercase consonants over a 2160 ms interval, followed by either a short delay of 500 ms or a maintenance (M) phase of 6480 ms. In the retrieval (R) phase, participants were probed with a single lowercase consonant and had 2160 ms to judge whether the probe letter corresponded to one of the letters in the encoding set by pressing either a *yes* or *no* response button. This was followed by a 6480 ms intertrial interval (ITI) for ER trials or a 500 ms ITI for EMR trials so that both trial types were equal in length (11300 ms). Participants performed a total of four conditions (3-letter ER, 6-letter ER, 3-letter EMR, and 6-letter EMR) in a randomized block design over two sessions. Each block consisted of four trials of a particular condition with a total of six blocks (24 trials) allocated to each condition.

For each memory load condition, we computed individual participants' “RT savings” due to the maintenance interval by subtracting each participant's RT in the EMR condition from their RT in the ER condition (RT savings = RT_ER_ – RT_EMR_). A measure of “accuracy savings” was computed in the same way as RT savings, *mutatis mutandis* (Accuracy savings = Accuracy_ER_ – Accuracy_EMR_). For the ER and EMR conditions, we computed individual participants' “RT slope” due to the load condition by subtracting each participant's average RT for low-verbal-load trials from the average RT of high-verbal-load trials and dividing by the load difference (e.g., for the EMR condition, RT slope = [RT_6-letter EMR_ – RT_3-letter EMR_]/3).

Stimuli were generated from a computer (Macintosh G3, Apple Computer, Cupertino, CA) using Psyscope 1.2.1 and back-projected onto a screen located above the subject's neck via a magnet-compatible projector. Stimuli were viewed from a mirror mounted above the subject's head. The sequence of the presentations of the stimuli was synchronized with the imaging sequence of the scanner.

#### fMRI Methodology

Imaging was performed with a 1.5T whole-body MRI scanner (General Electric Medical Systems Signa, Rev. 5.3). A custom quadrature receive-only birdcage head coil was used. Head motion was minimized using a bite-bar formed to the participant's dental impression. A T2* sensitive gradient echo spiral sequence [24] was used for functional imaging with parameters of TR = 2160 ms, TE = 40 ms, flip angle = 83°, FOV = 20 cm, inplane resolution = 3.125 mm^2^, and sampling interval = 2.16 s. Sixteen 7-mm thick slices with a 0-mm inter-slice interval were acquired in the horizontal plane of the Talairach and Tournoux atlas [25] covering the whole brain.

#### fMRI Analysis

Image analysis was performed by transferring the raw data to a Linux machine. A gridding algorithm was employed to resample the raw data onto a Cartesian matrix prior to processing with 2D FFT. Each subjects' functional images were motion-corrected and normalized using SPM99 (Wellcome Department of Cognitive Neurology, London, England), interpolated to 2×2×4 mm^3^ voxels and spatially smoothed with a Gaussian filter (8 mm FWHM). Differences in global signal were removed. Contrast images were created with a random-effects model from which group data were generated. Activation maps were created in SPM99 (http://www.fil.ion.ucl.ac.uk/spm) implemented in MATLAB (Mathworks, Inc., Sherborn). Data are reported for activation that survived a statistical threshold of *p*<.001 (uncorrected). Relationships between behavioral data and fMRI activation were investigated by identifying voxels that correlated strongly (p<.001, uncorrected)) with behavioral regressors across subjects.

### Experiment 2

#### Participants

In the second experiment, a different set of twelve subjects (5 female and 7 male right-handed native-English speakers, mean age = 20.6 yrs) were scanned via fMRI. All participants were undergraduate students from Stanford University and each provided a written consent which was approved by the Institutional Review Board.

### Behavioral Task

Subjects in *Experiment 2* maintained different numbers of letters on different trials (see Figure 1B). In the encoding phase of each trial, participants encoded three, four, five, or six uppercase consonants over a 2160 ms (1 frame) interval, followed by a maintenance phase of 6480 ms (3 frames). In the retrieval phase, participants were probed with a single lowercase consonant and had 2160 ms (1 frame) to judge whether the probe letter corresponded to one of the letters in the encoding set by pressing either a *yes* or *no* response button. This was followed by a 10800 ms (5 frames) ITI. The total duration for each trial was 21600 ms (10 frames). Stimuli were presented using Psyscope software on a Macintosh platform, and subjects' responses and reaction times were recorded. All subjects performed a total of 96 trials over four successive scans with 24 trials per letter load. Prior to scanning, all subjects received a battery of paper and pencil tests including the Digit Span Forward task (DF) outside the scanner.

#### fMRI Analysis

Data collection methods were identical to those used in *Experiment 1*. Data analyses methods were also identical to those described for *Experiment 1* except for post-processing differences necessary for the analysis of single-trial data, which are described below in detail. To ensure valid comparisons between experiments, both *Experiment 1* and *Experiment 2* were analyzed using random-effects models. Reconstructed images were analyzed using SPM99 implemented in MATLAB (Mathworks, Inc., Sherborn). Images were motion-corrected, normalized, spatially smoothed, and filtered as in *Experiment 1*.

An event-related approach was used to model Encoding, Maintenance, Retrieval and the intertrial interval. Regressor functions were generated by convolving a boxcar corresponding to each event with the canonical hemodynamic response function Incorrect trials were excluded. Contrasts between the phase of interest (Encoding, Maintenance, or Retrieval) and the intertrial interval were computed. Group data were analyzed via a random effects analysis. Statistical parametric maps were created for each contrast by transforming T-maps to normal Z-distributions. Data are reported for activation that survived a standard statistical threshold for event-related studies (p<.001 uncorrected). Relationships between behavioral data and fMRI activation were investigated by identifying voxels that correlated strongly (p<.001, uncorrected) with behavioral regressors across subjects.

## Results

### Experiment 1

#### Behavioral Data

Subjects' RTs (±SD) in the block design experiment were 987±43.9 ms in the 3-letter EMR condition, 994±57.0 ms in the 3-letter ER condition, 1167±49.6 ms in the 6-letter EMR condition, and 1149±30.2 ms in the 6-letter ER condition. Participants were faster in the 3-letter than in the 6-letter condition; the main effect of load on reaction time (RT) was significant (F[1,11] = 14, p<.004, MSe = 3.37). Participants' RTs were equivalent in the ER and the EMR conditions; the main effect of maintenance interval was not significant, and the memory load by maintenance interval interaction was also not significant.

These results suggested that the length of the maintenance interval had no effect on processing speed. We investigated these results further by taking individual capacity differences into account when assessing the effect of the maintenance interval. Individual differences in capacity were assessed using each participant's Digit Span Forward (DF) score. We then correlated each participant's DF score with their “RT savings”, which is a measurement of how efficiently the participant used the maintenance interval to improve retrieval rate (see Experimental Procedures). The results of this analysis indicated that RT savings was affected by maintenance duration in the 6-letter condition. There was a significant positive correlation between participant's RT savings in the high load condition and their DF score (r = 0.58, t = 2.12, *p* = .05) suggesting that higher RT savings are associated with higher capacity. No such correlation, however, was found in the low load condition between RT savings and capacity. The correlations between total RT (participants' average RT for a particular load) and capacity was not significant.

Subjects' mean percent accuracy (±SD) in the block design experiment was 95±5.0 in the 3-letter EMR condition, 93.5±10.9 in the 3-letter ER condition, 81.6±15.3 in the 6-letter EMR condition, and 91.7±8.4 in the 6-letter ER condition. Analysis of accuracy showed that there was a main effect of load (F[1, 11] = 14.8, *p*<.003, MSe = .07) such that participants were more accurate in the 3-letter condition, a main effect of maintenance interval (F[1, 11] = 6.6, *p*<.03, MSe = .02) such that participants were more accurate with a shorter delay period, and an interaction between load and maintenance interval (F[1, 11] = 6.9, *p*<.03, MSe = .04) such that the accuracy differed significantly between the ER and EMR conditions for the 6-letter load but not for the 3-letter load. However, there was not a significant correlation between accuracy and capacity, or between “accuracy savings” (see Experimental Procedures) and capacity.

To examine the relationships between participants' processing rate, WM capacity, and maintenance interval duration, we performed separate linear regression analyses of participants' DF score and their speed in the EMR and ER conditions as measured by “RT slope” (see Experimental Procedures). There was a stronger negative correlation and a steeper slope between DF scores and RT slope in the EMR condition (slope = −26.27, r = −0.77, t = 3.64, p = .004) than in the ER condition (slope = −19.011, r = −0.61, t = 2.30, p = .042). Thus, it appears that for participants' RT slopes, capacity scores accounted for more variability in the EMR than in the ER condition. A similar analysis was performed to examine the relationship between capacity and accuracy slope, but no significant correlations existed for either the EMR or ER conditions. Furthermore, in order to determine whether the relationships between capacity and RT savings and slope could be explained by a possible speed-accuracy trade-off, we performed correlational analyses to assess the relationship between RT and accuracy scores. The results revealed only weak correlations for speed and accuracy for each of the four conditions (3-letter EMR, 3-letter ER, 6-letter EMR, 6-letter ER), none of which achieved significance.

These behavioral results suggest that participants were utilizing their capacity resources during the maintenance interval of the higher load condition, as a greater amount of the variability in individuals' slopes could be accounted for by capacity, and those individuals with greater capacity benefited with greater RT savings specifically due to faster retrieval rates associated with the longer maintenance interval. Participants with lower capacity measures suffered performance decrements as their RT worsened in the EMR condition perhaps due to their inability to utilize capacity resources during the maintenance interval. Thus, WM capacity benefits WM speed, or retrieval rate, specifically during the high load condition, but did not correlate with behavioral measures such as accuracy or total reaction time.

#### fMRI Data

**Figure 2:**
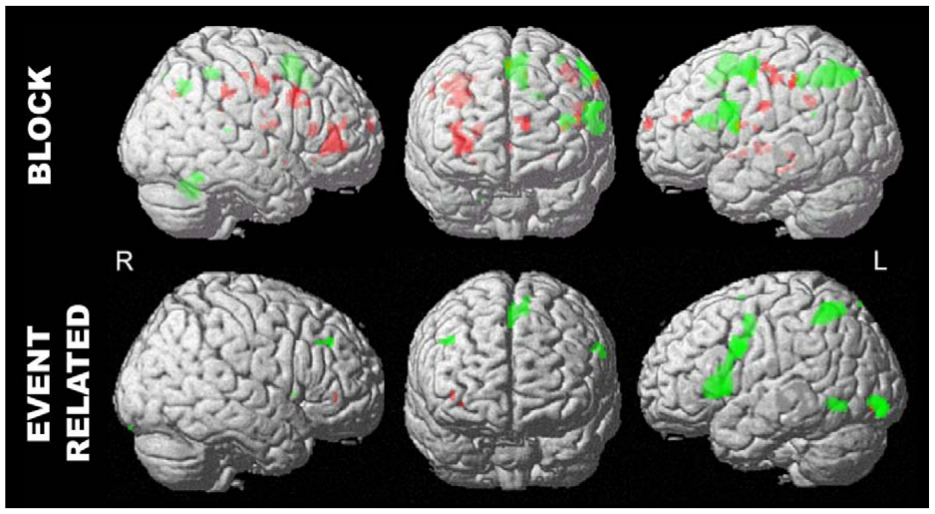
FMRI results of block-designed *Experiment 1* (upper row) and event-related designed *Experiment 2* (lower row). For *Experiment 1* activation maps represent significant task-related activation associated with the high-load maintenance interval (6-letter EMR condition compared to the 6-letter ER condition). For *Experiment 2*, activation maps represent significant task-related activation associated with the high-load maintenance interval (6-letter maintenance interval compared to the ITI). For all images, activity common to all participants is color-coded in green and activity varying with individual differences in capacity is color-coded in red. Images show regions that were significantly more active (p<.001, uncorrected).

**Figure 3:**
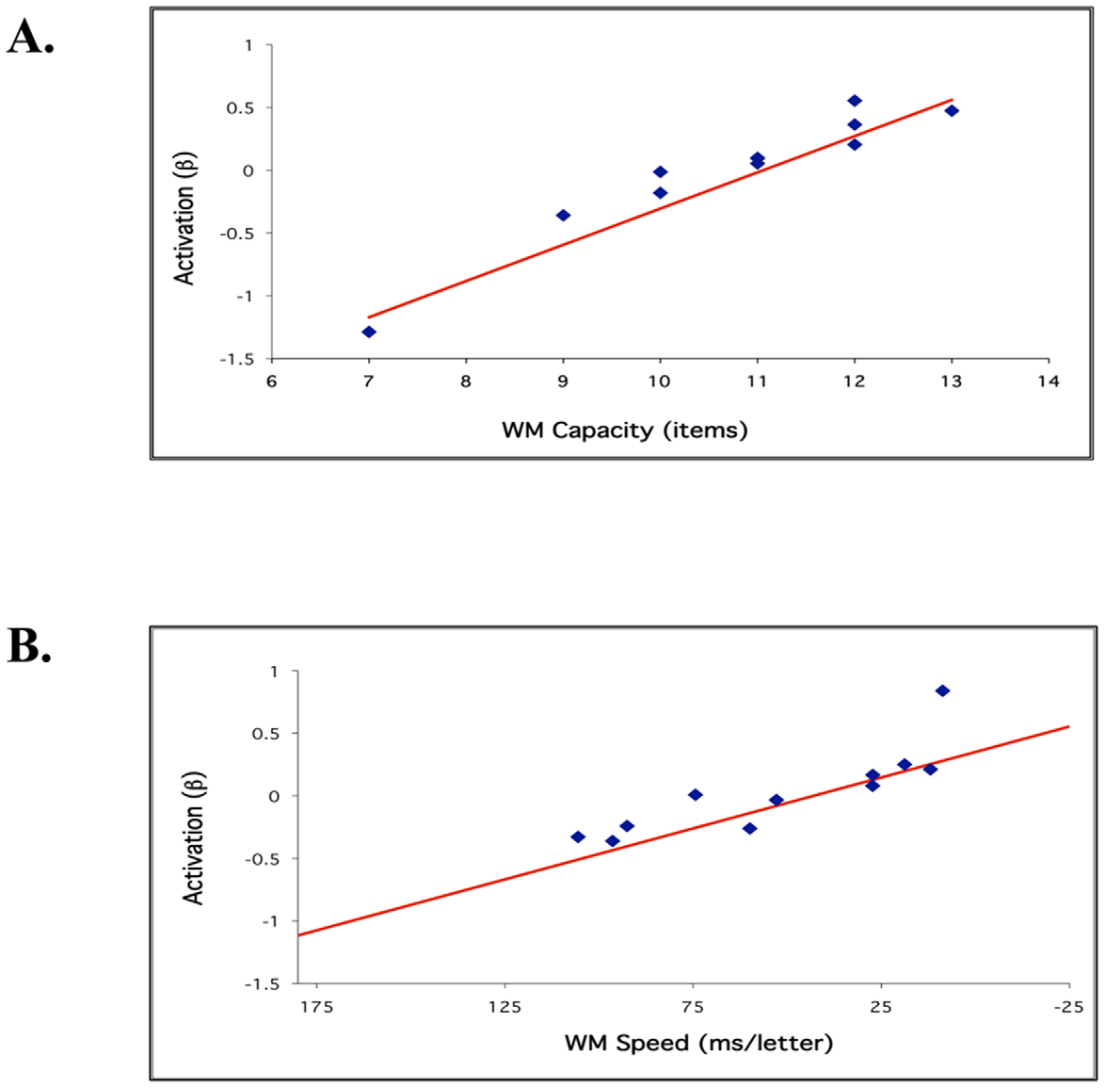
Correlations between PFC activation and behavioral results. (A) From *Experiment 1*, fMRI activation associated with high-load maintenance (6-letter EMR compared to 6-letter ER) in right PFC BA 10/46 vs. WM capacity (as measured by DF score). Participants with greater capacity showed greater activation in the right PFC region. The roi for figure 3a was the right 10/46 area activity which varied with individual differences in capacity as color-coded in red in Figure 2a. (B) From *Experiment 2*, fMRI activation associated with high-load maintenance (6-letter maintenance interval compared to ITI) in right PFC BA 10/46 vs. WM speed (as measured by RT Slope). Note that x-axis is reversed to illustrate that WM speed increases from left to right. Participants with faster processing rates showed greater activation in the right PFC region. The roi for figure 3b was the right 10/46 area activity which varied with individual differences in speed as color-coded in red in Figure 2b.

Results from *Experiment 1* indicated that during performance of the high-load EMR condition (compared to the high-load ER condition), participants demonstrated increased task-related activity in a predominantly left hemisphere network, consisting of frontal, parietal, and temporal cortex, a network active among all participants regardless of their WM capacity (Figure 2, upper row, shown in green). There were other regions, however, that demonstrated activity proportional to individual capacity scores. Correlational analyses revealed that individuals with greater capacity (as measured by the DF task) showed proportionate recruitment of right frontal regions (with major foci of activity in Brodmann Area 10/46) when maintaining high verbal load (Figure 2, upper row, shown in red). Figure 3A illustrates the positive correlation between PFC recruitment (as measured by the parameter estimate, β) and capacity scores of individual participants.

Consistent with this result, we observed that participants above a capacity score threshold of 11.0 (DF score range 7–13, average 10.7) demonstrated positive RT-savings, i.e., these participants were able to improve RT when presented with a longer maintenance interval which could be attributable to the utilization of capacity resources as demonstrated by their greater activity in right PFC. Those with capacity scores less than this threshold, however, demonstrated negative RT-savings which, alternatively, may be due to the under-utilization of capacity resources during the maintenance period. In addition, there was a positive relationship between capacity-based neural activity in right PFC and working memory speed as measured by RT slope (r = .67, t = −2.55, p = .014).

### Experiment 2

The results of *Experiment 1* suggested that a processing speed benefit was conferred upon participants to the extent that they performed additional processing (governed by right PFC) which most likely occurred during the delay interval. It is possible that the increased PFC activity is attributable to differences in other periods of the block-design paradigm (ITI) but only during the extended maintenance interval was additional processing required. To examine whether processing speed is influenced by other brain regions or task periods, and to replicate our results without the assumption of cognitive subtraction, we performed another experiment using single-trial methodology and parametrically varying memory load.

Analysis of these data allowed us to combine the results of the present experiment with those of *Experiment 1* and permitted examination of “capacity-based regions” (brain regions that show increased activity with increases in individual participants' memory spans) and “speed-based regions” (brain regions that show increased activation with increases in individual participants' retrieval speed). These results permit more precise inferences to be drawn regarding the neural basis for speed-capacity relations in human performance.

#### Behavioral Data

Subjects' RTs (±SD) in the single trial experiment were 1103.3±44.3 ms in the 3-letter condition, 1151.4±41.0 ms in the 4-letter condition, 1197.3 ms±42.4 ms in the 5-letter condition, and 1264.6±53.1 ms in the 6-letter condition. There was a main effect of load on reaction time (F[3,11] = 15.4, *p*<.0001, MSe = 61330).

Subjects' mean percent accuracy (±SD) were 95.5±1.6% accurate in the load 3-letter condition, 95.5±1.7% accurate in the load 4-letter condition, 84.3±2.9% accurate in the 5-letter condition, 92.0±1.5% accurate in the 6-letter condition. There was a main effect of load on accuracy (F[3,11] = 12.9, *p*<.0001, MSe = 364).

#### fMRI Data

The same left hemisphere network of frontal, parietal, and temporal regions that was shown in the block-design experiment was active among all participants in maintaining verbal information in the single trial experiment for the 6-letter memory load condition (Figure 2, lower row, shown in green). To test our hypothesis that PFC recruitment is associated with RT, we correlated participants' maintenance-related activity in PFC (versus the ITI) with their RT. These data indicated that individuals with faster retrieval rates more heavily recruited a PFC region (corresponding to BA 10/46) during the 6-letter memory-load condition (PFC activity in the 3-, 4-, and 5-letter conditions did not vary with retrieval rates).

Consistent with this observation, there was a positive correlation between individual participants' working memory speed and their neural activity in this area during the maintenance interval (Figure 3B). In line with our prediction that processing speed is supported by prefrontal regions, we observed that a retrieval rate of ∼55 ms/letter and faster led to non-negative parameter estimates for activation in right PFC, conforming our brain-activity predictions from the participants' retrieval behavior. In addition, there was a positive correlation between individual participants' capacity and their neural activity in right BA 10/46 during the maintenance interval (Figure 2, lower row, shown in red), replicating the results from experiment 1.

## Discussion

The left hemisphere network of activity involved in maintaining verbal information in WM is in agreement with numerous studies [16]–[18], [20], [21], [26]. This domain-specific network was utilized by all participants (despite their differing degrees of capacity) to maintain a high verbal WM load (i.e., 6 letters). The additional right PFC regions, utilized by individuals with greater WM capacity, have been implicated in domain independent (i.e., both spatial and non-spatial) processing in numerous studies (e.g., [27]). The increased capacity and faster RT in individuals in which this additional activation was observed may be afforded by the activation of these domain independent regions.

What might be the role of these domain-independent regions in increasing WM capacity? One possibility is that these activations reflect binding or chunking of to-be-remembered information in the service of increasing WM capacity. This idea is supported by other studies showing activation of these regions in manipulation of to-be-remembered information (e.g., [28]) and in integration of multiple forms of information in WM [16], [18], [29]–[31].

In behavioral data from our first experiment we observed relationships between participants' capacity (measured by DF score) and RT savings. Furthermore, the neuroimaging data indicated a positive relationship between neural activity in a right PFC region and WM speed (measured by RT slope). These results suggest that some participants benefited from the delay interval as indicated by (1) the positive correlation between participants' DF scores and their delay-related RT savings, and (2) the stronger negative correlation and steeper slope in the regression of RT slope and DF score in the EMR than in the ER condition.

Lesion studies conducted by Prabhakaran and colleagues [32] also support the findings that the right PFC plays a role in spatial and verbal WM tasks, providing evidence for the domain-independent nature of these regions. In this study, we showed that patients with lesions in right hemisphere cortical regions showed deficits on the spatial and verbal WM task compared to patients with lesions in left hemisphere cortical regions who showed deficits only on the verbal task (in the majority of these patients (64%), stroke affected PFC). The study is described in depth elsewhere [32] and we have conducted a reanalysis of the study for validating brain-behavior relationships (see Text S1 for details). In addition, our results suggest that these regions' ability to influence behavior is secondary to the capacity resources available. In our reanalysis we examined the behavioral performance (accuracy, encoding time, and RT to evaluate each of the sub-components of the WM task) in these patients with respect to their individual span measures. Vascular lesion patients classified as having high spatial span tended to be more accurate across all memory loads for the spatial WM task than patients with low spatial span (Table S2). In addition, at the low memory load, vascular lesion patients with high spatial span had significantly less deficits in accuracy and tended to have less deficits in RT compared to those with low spatial span for the spatial WM task. At the high spatial load, patients with high spatial spans tended to have less deficits in RT than low spatial span patients for the spatial WM task. Likewise, patients with high digit spans were significantly more accurate across all loads of the verbal WM task (Table S3). At the low verbal load, patients with high digit span tended to have fewer deficits in encoding time than those with low digit span, whereas at the high verbal load patients with high digit span tended to be to be more accurate than low digit span patients. These results suggest that vascular insults that negatively affect span measures also negatively affect WM accuracy and processing speed, providing further evidence for the interdependence of these measures (see also Tables S4, S5). In addition, when stroke patients were divided by age, only old left stoke patients had deficits in RT at the low verbal load, while both old left and old right stroke patients had deficits in RT at the high verbal load (Table S6). In contrast, young left stroke patients had a trend of compromised RT at the high verbal load only. This is consistent with the hypothesis that older individuals must recruit additional domain-independent resources for tasks due to declining structural or functional fidelity [33]. Together, this lesion evidence converges with our fMRI results, indicating an essential role for right PFC in multimodal or modality-independent WM and that these regions' ability to influence behavior is secondary to the capacity resources available.

Results from the neuroimaging and lesion experiments suggest that the additional right PFC recruitment in high-span participants during the maintenance interval reflects cognitive mechanisms that improve the quality of maintained information, rendering it more available for subsequent retrieval. It is worth noting that only participants with capacities greater than 10 (as measured by DF scores) showed right PFC activation, as indicated by the fact that parameter estimates, β, were in the positive range for these participants (Figure 3A). This suggests that a processing rate benefit was observed in participants to the extent that they showed right PFC recruitment. The single-trial method implemented in the *Experiment 2* permitted the examination of different phases of our memory paradigm (i.e. encoding, maintenance, retrieval). Thus, we could assess the extent to which participants' retrieval rate was influenced by neural activity during the maintenance interval. The results of this analysis revealed activity in right PFC regions during the maintenance phase that correlated with individual participants' retrieval processing rates. Similar “subsequent-memory effects” have been observed in dorsolateral PFC (DLPFC) in other WM studies [34]. DLPFC may play a critical role in memory processes that permit efficient memory maintenance and retrieval. It may be that DLPFC regions function in WM to consolidate, or bind, information in the service of overcoming capacity limits [16]–[18], [26]–[28], [34], [35].

**Figure 4:**
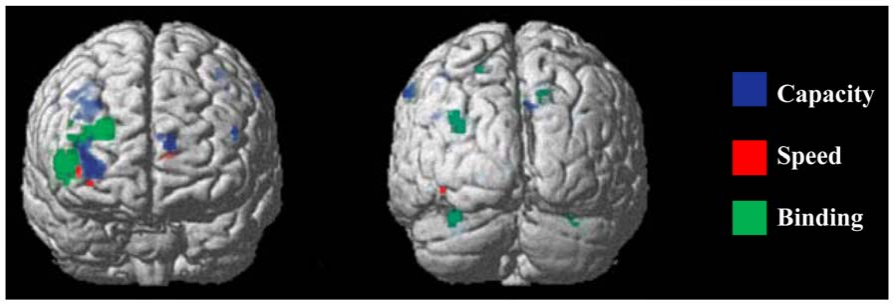
Overlap analysis. *Experiment 1* results showing high memory load retention interval activity varying with individual participants' capacity (i.e., DF score) is coded in blue - Capacity. *Experiment 2* results showing high memory load retention interval activity varying with individual differences in retrieval rate is coded in red - Speed. Previous study (16) showing retention interval activity while maintaining bound (vs. unbound) information in memory is coded in green - Binding. The image shows 16 of 193 voxel overlap between speed and capacity maps (p = .05) and 100% overlap between speed and binding as well as capacity and binding (p = .05). Individual color maps voxelwise threshold is p<.001, uncorrected.

Our behavioral results showing that high-span participants' retrieval rates were increased with longer maintenance intervals suggest that participants utilized the delay interval between encoding and retrieval to carry out binding operations that effectively reduce WM load. We sought support for the notion that high-span participants' increased right DLPFC activity reflected consolidation processes by performing an overlap analysis with the present data and data from an earlier study that explicitly required information binding [18]. In the overlap analysis, we superimposed “capacity-based regions” (i.e., normalized regions whose activity varied with DF score), “speed-based regions” (i.e., normalized regions whose activity varied with retrieval speed), and binding activation data from the previous study. Figure 4 illustrates the overlap between capacity-based, speed-based, and binding activation regions. The intersection between these three maps provides support for the notion that increased capacity resources results from strategic consolidation of to-be-remembered information in PFC, resulting in increased processing speed. Although our experiments were not designed to address the specific issue of chunking, our interpretation is supported by studies that have reported activations in the right lateral PFC when the subjects were engaged in consolidation of the to-be-remembered items (e.g., [16], [17], [36]). Using mathematical stimuli and carefully designed control experiments, Bor and Owen [36] demonstrated a link between chunking and the bilateral lateral prefrontal cortices. An alternative explanation is that the participants engaged in an articulatory subvocal rehearsal process during the maintenance period; however, there is evidence that the articulatory loop engages areas such as the SMA, Broca's area, and possibly even the cerebellum, none of which were actively recruited during the maintenance phase in the two experiments reported here.

Evidence from the validation study with stroke patients also supports the notion that the right PFC region is associated with memory consolidation. Patients with lesion in the right PFC areas showed deficits in accuracy and retrieval speed in both spatial and verbal WM, suggesting that the right frontal patients had problems with maintaining and retrieving the encoded items in an efficient manner.

These results have a number of implications. First, they suggest that individual differences in the ability to implement binding or chunking strategies in WM may underlie performance differences between individuals in both their capacity and speed. Moreover, the brain basis of this capacity construct involves flexible recruitment of domain-independent regions (in right DLPFC) to effectively expand the storage capacity of domain-dependent regions (in a left-hemisphere network for verbal material).

Determination of the true capacity limits of short-term storage has been considered essential to understanding human mental processes [22], [37]. Accordingly, the mechanisms available to individuals to overcome capacity limits have been closely studied. The relative availability of these mechanisms between individuals appears to reflect WM capacity differences. The present results suggest that some individuals, more than others, utilize domain-independent PFC-based resources.

“Exhaustive search” [1] (that is, search of the entire memory-set independent of the target's list location or presence) has been posited as the mechanism underlying the near-linear relationship between memory set-size and reaction time. Other self-terminating mechanisms (that is, search that terminates when the target is located) have also been advanced [38]. Three features of our results suggest more support for exhaustive than self-terminating memory-search mechanisms. First, our behavioral results indicated minimal RT slope differences between yes vs. no probes. Second, we found no effect of probe position on RT. Third, participants who showed activity in domain-independent binding regions apparently had to search fewer items, as they showed reduced RT slopes compared to those who did not show such activity. Moreover, prior evidence from our laboratory [18] suggests that retrieval of individual items from a memory-set is faster and more accurate when items can be searched as a bound unit than when they cannot, which indicates the efficiency of exhaustive search.

Taken together the results of the present study suggest that common mechanisms, located in dorsal PFC regions of the right hemisphere, mediate memory consolidation processes. The benefits to individuals that implement these strategic processes are increases in WM capacity and processing speed. Benefits may in turn accrue to higher cognitive processes that depend on WM such as language, text comprehension, and reasoning. Thus, this mechanism may provide the basis for Spearman's original observation of ubiquitous positive correlations among diverse measures of mental ability [39].

## Supporting Information

Text S1 Detailed methods and reanalysis procedures for lesion data from Experiment 3.
(DOC)

Table S1 Mean (standard deviation) accuracy for participants in span tasks.
(DOC)

Table S2 Percentage of high- and low-spatial-span vascular patients demonstrating deficits during the spatial working memory task. Low spatial load refers to 1- and 2-location conditions; high spatial load refers to 3- and 4-location conditions. The *p*-value represents significance of the between-groups (high- vs. low-spatial-span) *T* test.
(DOC)

Table S3 Percentage of high- and low-digit-span vascular patients demonstrating deficits during the verbal working memory task. Low verbal load refers to 3- and 4-letter conditions; high verbal load refers to 5- and 6-location conditions. The *p*-value represents significance of the between-groups (high- vs. low-digit-span) *T* test.
(DOC)

Table S4 Mean accuracy, encoding time, and retrieval time for right stroke, left stroke, and TIA patients during the spatial working memory task. The *p*-value represents significance of the between-groups *T* test (*either* right-stroke vs. TIA *or* left-stroke vs. TIA). Note: *p<.05.
(DOC)

Table S5 Mean accuracy, encoding time, and retrieval time for right stroke, left stroke, and TIA patients during the verbal working memory task. The *p*-value represents significance of the between-groups *T* test (*either* right-stroke vs. TIA *or* left-stroke vs. TIA). Note: *p<.05; **p<.01; ***p<.001.
(DOC)

Table S6 Percentage of young (age<50 yrs) and old (age = 50+ yrs) vascular patients demonstrating reaction time deficits during the verbal working-memory task. Low verbal load refers to 3- and 4-letter conditions; high verbal load refers to 5- and 6-letter conditions. The *p*-value represents significance of the between-groups *T* test (for each age group, *either* right-stroke vs. TIA *or* left-stroke vs. TIA). Note: *p<.1; **p<.05; ***p<.005.
(DOC)
