# Supplementary material for: Capacity-Speed Relationships in Prefrontal Cortex
Source: PLoS One. 2011 Nov 23;6(11):e27504. doi: 10.1371/journal.pone.0027504 (PMC3223164; doi:10.1371/journal.pone.0027504)
Supplement: Text S1 — Detailed methods and reanalysis procedures for lesion data from Experiment 3. (DOC) [file pone.0027504.s001.doc]

***Experiment 3***

**Participants**

Participants were 40 right-handed patients with acute ischemic stroke studied within three days of symptom-onset. Twenty-seven right-handed patients with transient ischemic attack (TIA; defined as resolution of symptoms within 24 hours and stroke excluded by MRI) were enrolled to serve as hospitalized controls with similar ages and other demographic factors, vascular risk factors, and stressors of hospitalization to those of the stroke patients. Patients with the following conditions were excluded: (1) premorbid dementia, neurological, or psychiatric disease; (2) contraindication for MRI; (3) diminished level of consciousness; (4) lack of premorbid proficiency in English; (5) visual field deficits as determined by medical records and bedside examination; (6) motor deficits precluding performance of computer tasks; (7) language deficits that interfered with task comprehension; or (8) left neglect, defined by >10% errors on the left half of the page in line cancellation. Stroke was defined as acute infarct on DWI and/or hypoperfusion on PWI with corresponding neurological deficits.

Stroke patients were categorized as cortical strokes (right or left) or non-cortical strokes (basal ganglia, thalamus, internal capsule, white matter only, brain stem, or cerebellar), and then further classified as left or right hemisphere depending on stroke location. For the age comparison, patients were defined as younger (<50 yrs) or older (50+ yrs). For age-split analysis, non-cortical stroke patients were included in order to increase the number of patients in the analysis.

**Behavioral Task**

Participants performed subject-paced computerized verbal and spatial WM tasks with their dominant hand, unless they had arm or hand weakness such that they preferred to use the non-dominant hand. On each of 48 trials, they (1) encoded 3, 4, 5, or 6 to-be-remembered letters or 1, 2, 3, or 4 locations, (2) maintained these letters (or locations) across a 5-second unfilled delay, and (3) indicated whether or not a single probe letter (or location) was part of the memory set. For the verbal task, the to-be-remembered letters and probe letters were shown in different cases, so that the subjects encoded the verbal identity of letters rather than their visual appearance. They also completed digit and spatial span tasks. The digit span task required subjects to immediately repeat random sequences of digits increasing by one digit in length. The test was stopped after two consecutive incorrect responses, and the previous span length was recorded as the subject’s span. Similarly, the spatial span task required subjects to tap random sequences (of increasing length) of blocks on a page immediately following the examiner’s tapping. Participants were required to perform the span tasks in forward and backward sequence relative to the examiner, and a total score summing the performance length in each was noted. Using the resulting span measures, vascular patients (TIA and stroke) were split into high and low span groups based on their digit and spatial span scores using a median split. To contrast performance in the Sternberg-WM tasks, deficits in encoding time, retrieval time, and accuracy (percent correct) were compared between groups. A pooled WM score was calculated by summing the deviations from the mean, as measured by z-scores, for encoding time, retrieval time, and accuracy. WM deficits were defined as having a pooled score more than two standard deviations from the mean pooled scores taken from 20 normal volunteers. Frequencies of deficits were compared between control and stroke groups using nonparametric contingency tables. Due to the low n for some comparisons, Fisher’s Exact Tests were used when chi-square tests were not appropriate (i.e. when the expected values were <5).

**fMRI Analysis**

The following MRI sequence were used on all patients as part of the Johns Hopkins Acute Stroke Protocol: diffusion weighted imaging (DWI) and perfusion weighted imaging (PWI), axial T2, fluid attenuated inversion recovery (FLAIR), gradient echo (GE), and apparent diffusion coefficient (ADC) maps. The reported analyses used DWI (after confirming the acuity of the lesion as dark on ADC maps) and PWI (co-registered to T2 to provide anatomical boundaries that are less visible on PWI). Whole brain coverage was obtained by DWI and PWI scans of 5-mm thick slices. Volumes of tissue dysfunction on DWI and/or PWI were calculated using the Image J program. If a patient had imaging abnormalities on both DWI and PWI, the larger calculated volume was used. Volume of PWI abnormality was measured on time to peak maps, defining hypoperfused tissue as voxels where there was >4 seconds delay in time to peak arrival of contrast, relative to the homologous region in the normal hemisphere.
