# Supplementary material for: Capacity-Speed Relationships in Prefrontal Cortex
Source: PLoS One. 2011 Nov 23;6(11):e27504. doi: 10.1371/journal.pone.0027504 (PMC3223164; doi:10.1371/journal.pone.0027504)
Supplement: Table S1 — Mean (standard deviation) accuracy for participants in span tasks. (DOC) [file pone.0027504.s002.doc]

|  | **Experiment 1** (n = 12) | | **Experiment 2** (n = 12) | |
| --- | --- | --- | --- | --- |
| Digit – Symbol | 76.7 (9.5) |  | 78.7 (7.0) |  |
| Digit Span - Forward | 10.7 (1.7) |  | 10.6 (1.8) |  |
| Digit Span - Backward | 10.2 (1.8) |  | 10.1 (2.5) |  |
| Alpha Span | 8.1 (1.6) |  | 7.5 (1.7) |  |
| Listening Span | 33.5 (11.3) |  | 38.8 (10.1) |  |
