# Supplementary material for: Capacity-Speed Relationships in Prefrontal Cortex
Source: PLoS One. 2011 Nov 23;6(11):e27504. doi: 10.1371/journal.pone.0027504 (PMC3223164; doi:10.1371/journal.pone.0027504)
Supplement: Table S2 — Percentage of high- and low-spatial-span vascular patients demonstrating deficits during the spatial working memory task. Low spatial load refers to 1- and 2-location conditions; high spatial load refers to 3- and 4-location conditions. The p-value represents significance of the between-groups (high- vs. low-spatial-span) T test. (DOC) [file pone.0027504.s003.doc]

|  | **Measure** | **High Spatial Span** (n = 9) | | **Low Spatial Span** (n = 10) | | *p* |
| --- | --- | --- | --- | --- | --- | --- |
| **All Spatial Loads** | Accuracy | 0% |  | 40% |  | 0.087 |
| **Low Spatial Load** | Accuracy | 0% |  | 50% |  | < 0.05 |
| **Low Spatial Load** | Response Time | 33% |  | 80% |  | 0.069 |
| **High Spatial Load** | Response Time | 22% |  | 70% |  | 0.069 |
