# Supplementary material for: Capacity-Speed Relationships in Prefrontal Cortex
Source: PLoS One. 2011 Nov 23;6(11):e27504. doi: 10.1371/journal.pone.0027504 (PMC3223164; doi:10.1371/journal.pone.0027504)
Supplement: Table S3 — Percentage of high- and low-digit-span vascular patients demonstrating deficits during the verbal working memory task. Low verbal load refers to 3- and 4-letter conditions; high verbal load refers to 5- and 6-location conditions. The p-value represents significance of the between-groups (high- vs. low-digit-span) T test. (DOC) [file pone.0027504.s004.doc]

|  | **Measure** | **High Digit Span** (n = 6) | | **Low Digit Span** (n = 5) | | *p* |
| --- | --- | --- | --- | --- | --- | --- |
| **All Verbal Loads** | Accuracy | 17% |  | 100% |  | < 0.05 |
| **Low Verbal Load** | Encoding Time | 17% |  | 80% |  | 0.08 |
| **High Verbal Load** | Accuracy | 17% |  | 80% |  | 0.08 |
