# Supplementary material for: Capacity-Speed Relationships in Prefrontal Cortex
Source: PLoS One. 2011 Nov 23;6(11):e27504. doi: 10.1371/journal.pone.0027504 (PMC3223164; doi:10.1371/journal.pone.0027504)
Supplement: Table S4 — Mean accuracy, encoding time, and retrieval time for right stroke, left stroke, and TIA patients during the spatial working memory task. The p-value represents significance of the between-groups T test (either right-stroke vs. TIA or left-stroke vs. TIA). Note: *p<.05. (DOC) [file pone.0027504.s005.doc]

|  |  | **Right Stroke** (n = 13) | | **TIA**  (n = 20) | | **Left Stroke** (n = 10) | |
| --- | --- | --- | --- | --- | --- | --- | --- |
| **1-Location** | Accuracy | *0.67 |  | 0.76 |  | 0.79 |  |
|  | Encoding Time in ms | 5538 |  | 4353 |  | 5748.7 |  |
|  | Retrieval Time in ms | 5544.8 |  | 4805.4 |  | 5873.1 |  |
| **2-Location** | Accuracy | 0.68 |  | 0.67 |  | 0.72 |  |
|  | Encoding Time in ms | 5972.3 |  | 4901.1 |  | 4997.5 |  |
|  | Retrieval Time in ms | *5496.8 |  | 4221.7 |  | 6091.1 |  |
| **3-Location** | Accuracy | 0.68 |  | 0.72 |  | 0.75 |  |
|  | Encoding Time in ms | 6809 |  | 5965.7 |  | 6817.6 |  |
|  | Retrieval Time in ms | *6793.6 |  | 4225.1 |  | 5698.8 |  |
| **4-Location** | Accuracy | 0.59 |  | 0.61 |  | 0.68 |  |
|  | Encoding Time in ms | 7664 |  | 7254.2 |  | 8204.6 |  |
|  | Retrieval Time in ms | 5873 |  | 4868.8 |  | 6373.4 |  |
