# Supplementary material for: Capacity-Speed Relationships in Prefrontal Cortex
Source: PLoS One. 2011 Nov 23;6(11):e27504. doi: 10.1371/journal.pone.0027504 (PMC3223164; doi:10.1371/journal.pone.0027504)
Supplement: Table S5 — Mean accuracy, encoding time, and retrieval time for right stroke, left stroke, and TIA patients during the verbal working memory task. The p-value represents significance of the between-groups T test (either right-stroke vs. TIA or left-stroke vs. TIA). Note: *p<.05; **p<.01; ***p<.001. (DOC) [file pone.0027504.s006.doc]

|  | **Measure** | **Right Stroke** (n = 13) | | **TIA** (n = 19) | | **Left Stroke** (n = 13) | |
| --- | --- | --- | --- | --- | --- | --- | --- |
| **3-Letter** | Accuracy | *0.75 |  | 0.89 |  | 0.74 |  |
|  | Encoding Time in ms | *5862.0 |  | 4281.9 |  | *6234.2 |  |
|  | Retrieval Time in ms | *3665.2 |  | 2232.3 |  | *4167.0 |  |
| **4-Letter** | Accuracy | *0.74 |  | 0.88 |  | 0.76 |  |
|  | Encoding Time in ms | *7203.8 |  | 6334.6 |  | *8196.9 |  |
|  | Retrieval Time in ms | *3578.1 |  | 2241.4 |  | *3549.34 |  |
| **5-Letter** | Accuracy | **0.68 |  | 0.83 |  | ***0.61 |  |
|  | Encoding Time in ms | 8973.0 |  | 6334.6 |  | 10648.7 |  |
|  | Retrieval Time in ms | ***4516.5 |  | 2442.2 |  | ***4785.2 |  |
| **6-Letter** | Accuracy | 0.72 |  | 0.82 |  | **0.64 |  |
|  | Encoding Time in ms | 10522.8 |  | 7782.2 |  | 10418.6 |  |
|  | Retrieval Time in ms | ***3718.6 |  | 2443.6 |  | ***4199.8 |  |
