# Supplementary material for: Capacity-Speed Relationships in Prefrontal Cortex
Source: PLoS One. 2011 Nov 23;6(11):e27504. doi: 10.1371/journal.pone.0027504 (PMC3223164; doi:10.1371/journal.pone.0027504)
Supplement: Table S6 — Percentage of young (age<50 yrs) and old (age = 50+ yrs) vascular patients demonstrating reaction time deficits during the verbal working-memory task. Low verbal load refers to 3- and 4-letter conditions; high verbal load refers to 5- and 6-letter conditions. The p-value represents significance of the between-groups T test (for each age group, either right-stroke vs. TIA or left-stroke vs. TIA). Note: *p<.1; **p<.05; ***p<.005. (DOC) [file pone.0027504.s007.doc]

|  | | **YOUNG** | | | | | |  |  | **OLD** | | | | | | |  |
| --- | --- | --- | --- | --- | --- | --- | --- | --- | --- | --- | --- | --- | --- | --- | --- | --- | --- |
|  |  | **Right Stroke** (n = 2) | | **TIA** (n = 6) | | **Left Stroke** (n = 5) | |  |  | **Right Stroke** (n = 10) | | **TIA** (n = 13) | | | **Left Stroke** (n = 9) | |  |
| **Low Verbal Load** |  | 50% |  | 0% |  | 20% |  |  |  | 50% |  | | 15% |  | **67% |  | |
| **High Verbal Load** |  | 50% |  | 17% |  | *80% |  |  |  | ***80% |  | | 15% |  | *56% |  | |
